# Transparent Windows on Food Packaging Do Not Always Capture Attention and Increase Purchase Intention

**DOI:** 10.3389/fpsyg.2020.593690

**Published:** 2020-11-12

**Authors:** Xueer Ma, Xiangling Zhuang, Guojie Ma

**Affiliations:** Shaanxi Key Laboratory of Behavior and Cognitive Neuroscience, School of Psychology, Shaanxi Normal University, Xi’an, China

**Keywords:** transparent packaging, willingness to purchase, attractiveness, eye tracking, visual attention

## Abstract

Transparent windows on food packaging can effectively highlight the actual food inside. The present study examined whether food packaging with transparent windows (relative to packaging with food‐ and non-food graphic windows in the same position and of the same size) has more advantages in capturing consumer attention and determining consumers’ willingness to purchase. In this study, college students were asked to evaluate prepackaged foods presented on a computer screen, and their eye movements were recorded. The results showed salience effects for both packaging with transparent and food-graphic windows, which were also regulated by food category. Both transparent and graphic packaging gained more viewing time than the non-food graphic baseline condition for all the three selected products (i.e., nuts, preserved fruits, and instant cereals). However, no significant difference was found between transparent and graphic window conditions. For preserved fruits, time to first fixations was shorter in transparent packaging than other conditions. For nuts, the willingness to purchase was higher in both transparent and graphic conditions than the baseline condition, while the packaging attractiveness played a key role in mediating consumers’ willingness to purchase. The implications for stakeholders and future research directions are discussed.

## Introduction

When consumers stand in front of the shelves in a supermarket, food packaging attracts their attention, and plays an important role in shaping consumers’ food choice behavior. Food packaging not only protects food and extends the shelf life of food products, but also conveys the information like product attributes, price, and promotional messages (Hawkes, 2010). For customers, food packaging is a direct source to obtain food-related information such as the food category, brand, manufacturer, and expiration date, which form the basis of food decision-making. With the increase in market competition, food manufacturers are paying increasing attention to packaging design to maintain regular customers and attract new customers (Silayoi and Speece, 2007; Kuvykaite and Navickiene, 2009; Estiri et al., 2010; Simmonds and Spence, 2019). Opaque packaging is widely used in food packaging, especially packaging with product images. Imagery can capture consumer attention, provide information about the product and brand, and increase overall interest in the product, which promotes consumers’ purchase intentions (Simmonds and Spence, 2019). One emerging trend of packaging design involves transparent elements in food packaging. Packaging with transparent elements shows consumers the most authentic appearance of the food inside the packaging (Fernqvist et al., 2015). Compared with opaque packaging (with or without a product image), what superiority do packages with transparent elements have? What’s the role of transparent elements in shaping consumers’ feeling? How does it influence consumers’ purchase behavior? These questions have been partially answered by several previous studies.

In the review of visual factors influencing consumers’ visual hunger, Spence et al. (2016) pointed out that the visibility of attractive foods, or viewing pictures of desirable food, increases our visual hunger. This opinion is consistent with the “salience effect” hypothesis proposed by Deng and Srinivasan (2013). According to this hypothesis, the transparent packaging makes the food inside more conspicuous, which increases food consumption. However, such effects were modulated by food size and appearance. Deng and Srinivasan (2013) used foods like candies and cookies as experimental materials and examined the effects of food size, appearance, and packaging transparency on food consumption. They found that the salience effect was most apparent when small and visually attractive foods were presented with transparent packaging. However, the “monitoring effect,” which refers to transparent packaging enabling consumers to monitor their consumption better and thus eat less, was apparent when participants were shown large, unattractive foods. The researchers also found that seeing vegetables in a transparent package decreased the consumption of vegetables, which might be because vegetables were not regarded as tasty or attractive.

In order to systematically explore the relationship between packaging transparency and purchase intention, Simmonds et al. (2018b) classified packaging transparency into three categories: transparent with a visible product object (“window”), completely opaque with a product image (“graphic”), and completely opaque with no product image (“blank”). The researchers examined how packaging transparency affected consumer attitude, like expected tastiness, expected food quality, and expected freshness, as well as purchase intention. The materials included four kinds of food packaging pictures, used on cereal, boxed chocolates, dried pasta, and fresh fish. They found that participants preferred to buy foods with transparent packaging rather than opaque packaging. Participants also perceived food in transparent packaging to be fresher, tastier, and more innovative. The data suggest that seeing the food directly through the transparent window may induce the salience effect, resulting in higher hunger levels and food demand, leading to higher purchase intention (see also Billeter et al., 2012).

However, transparent packaging does not necessarily trigger positive feelings. Chandran et al. (2009) examined how packaging transparency affected the perceptions of food quality, product trust, and purchase intentions for both familiar and unfamiliar brands. The results showed that participants judged an unfamiliar product to be of higher quality and trustworthiness when it was in transparent packaging and preferred to pay more for it. In contrast, participants regarded familiar products with transparent packaging as being of lower quality, though participants did not show more distrust than toward the products with opaque packaging. Therefore, both product trust and product familiarity modulated the perception of food quality, which impacted purchase intention. Moreover, Riley et al. (2015) stated that transparent windows decrease, rather than increase, the perceived healthiness of the tested products, such as coffee, carrot soup, and carrot baby food. These sensory evaluations suggest that transparent packaging does not always induce salience effects.

Vilnai-Yavetz and Koren (2013) further examined how extra variables like perceived ease of use, aesthetics, and symbolism influence the evaluation of, and purchase intention toward, foods with transparent packaging. The researchers asked the participants to purchase mixed boiled vegetable meals with either an opaque wrapper showing a picture of the food, or a transparent plastic cover revealing the food inside. The product with the transparent cover had 30% lower sales than the product with opaque products. The evaluation tasks showed that consumers had more interest in buying products with opaque packaging. The transparent packaging evoked higher perceived ease of use (e.g., the package looks easier to open and seems to heat up easily) and lower perceived aesthetics (e.g., the package is unattractive) and symbolism (e.g., food quality does not look very good) than opaque packaging. Therefore, perceived aesthetics and symbolism also modulate the salience effect of packaging transparency. In the case of vegetables or vegetable meals, transparent packaging appears to have little superiority over opaque packaging in terms of food choice.

In sum, previous studies suggest that transparent packaging induces salience effects, such as triggering high levels of hunger and a higher willingness to purchase. However, transparent packaging may decrease several subjective feelings such as product quality, trustworthiness, aesthetics, and healthiness, and these effects are constrained by product categories and product familiarity (see the review by Simmonds and Spence, 2017). Therefore, more kinds of products should be tested to extend the knowledge about the role of packaging transparency. Additionally, the salience effect hypothesis predicts that transparent packaging will attract consumer attention, but the prediction is based on the literature of food-related attention bias studies using probe detection tasks. Eye-tracking approaches have not been used to test whether transparent packaging attracts consumer attention, and how this attraction modulates purchase behaviors. Therefore, the more objective method of eye tracking should be used to monitor consumer attention when evaluating food products with different packaging transparencies.

Currently, as technology develops to explore consumers’ visual processing behaviors in the real world, the eye-tracking approach is increasingly applied in the field of consumer behavior and marketing (Wedel and Pieters, 2007) to explore the impact of various packaging features on consumers’ visual attention and food choices (Piqueras-Fiszman et al., 2013; Bialkova et al., 2014, 2020; Husic-Mehmedovic et al., 2017; Fenko et al., 2018; García-Madariaga et al., 2019; Peschel et al., 2019; Ma and Zhuang, 2020). For instance, Piqueras-Fiszman et al. (2013) combined eye tracking and word association to assess novel packaging solutions. They found that certain elements of the product packaging attracted visual attention. In particular, consumers paid more attention to the photo on the package than the text in the same place. Consumers also preferred to buy the product with a photo rather than a message text on the packaging. According to the salience effect hypothesis (Deng and Srinivasan, 2013), transparent elements in packaging can attract consumer attention, subsequently increase their willingness to purchase.

In the literature about the relationship between attention and choice (Orquin and Mueller Loose, 2013; Van Loo et al., 2018), attention is a primary condition for the choice of corresponding objects. Some non-food studies showed that long processing time could predict preference for that stimulus (Maughan et al., 2007; Armel et al., 2008), and fixation time measures showed a similar trend when eye-tracking approaches were used (Gidlöf et al., 2017). In simulated shopping situations, the food product fixated on most had the highest probability of being chosen (Bialkova et al., 2014). However, the relationship between viewing time and preference formation may depend on experimental tasks. Wolf et al. (2018) found that longer viewing was associated with a higher likelihood of a positive evaluation in the self-paced exclusive evaluation (i.e., purchase intention “No” or “Yes”), but not in the self-paced non-exclusive evaluation (i.e., a Likert scale using 1–5 to indicate the attitude from dislike to like). Therefore, it is unclear whether more viewing time on transparent packaging leads to higher purchase intention. The present study not only tested the salience hypothesis for transparent packaging by using an objective eye-tracking approach, but also examined the relationship between attention and perceptual evaluation.

Following the study performed by Simmonds et al. (2018b), we used three packaging types: packaging with a transparent window (“transparent”), completely opaque packaging with a food image (“graphic”), and completely opaque packaging with a non-food image (“baseline”). This baseline is different from the blank condition used by Simmonds et al. (2018b), in that this study includes a match-sized non-food object to balance the visual differences of three packaging types. This control variable would make sure the differences among the three packaging types were caused by packaging types, rather than visual differences. The materials included packaging of the following three kinds of foods: nuts, preserved fruits, and instant cereals (see detailed information in the “Material” section). In the process of viewing food packaging, we not only recorded consumers’ eye movements, but also recorded their willingness to purchase the corresponding products, and the attractiveness evaluation of the corresponding packaging. Based on the following two important characteristics of the salience effect hypothesis (Deng and Srinivasan, 2013): (1) Salient food packaging elements can effectively attract the attention of consumers; (2) Attention attracted by salient elements will improve consumers’ willingness to purchase, two hypotheses were proposed:

*H1*: Transparent, rather than graphic, packaging can attract more attention (i.e., longer total time, larger number of fixations, and shorter time to first fixation of the transparent window) than packaging in the baseline condition.
*H2*: The eye movement measures reflecting salience effects are related to a willingness to purchase.

However, according to previous studies (Deng and Srinivasan, 2013; Vilnai-Yavetz and Koren, 2013), if product category regulated salience effects, the eye movement measures would show different results for different product categories. Using the eye-tracking approach, we expect the present study to provide insights on the role of transparent packaging and thus contribute to knowledge of food packaging design.

## Materials and Methods

### Participants

A power calculation performed using the G*power software suggests that at least 52 subjects per condition are needed to detect a medium effect size (most of effect sizes for WTP were larger than 0.3 in Simmonds et al., 2018b) of *d* = 0.8 with *α* = 0.05 for a one-way ANOVA analysis. In addition, a recent review on eye-tracking studies of nutrition label processing indicated that a small and convenient sampling could provide reliable findings on consumer behavior (Ma and Zhuang, 2020). In the review, there were 33% studies with sample size less than 60 participants and the smallest sample size was only 10 participants (Bialkova and van Trijp, 2011). Therefore, a convenient sample of 55 college students (30 females, ages between 18 and 21; 25 males, aged between 18 and 21) from Shaanxi Normal University in Xi ‘an, China, was paid to participate in the experiment. The participants had no color blindness, color weakness, or history of mental illness. They had normal or corrected-to-normal vision. Ten students were unable to conduct the formal experiment due to calibration or other eye-tracking problems. Thus, 45 participants (25 females and 20 males) were kept for final data analyses.

### Materials

**Figure 1:**
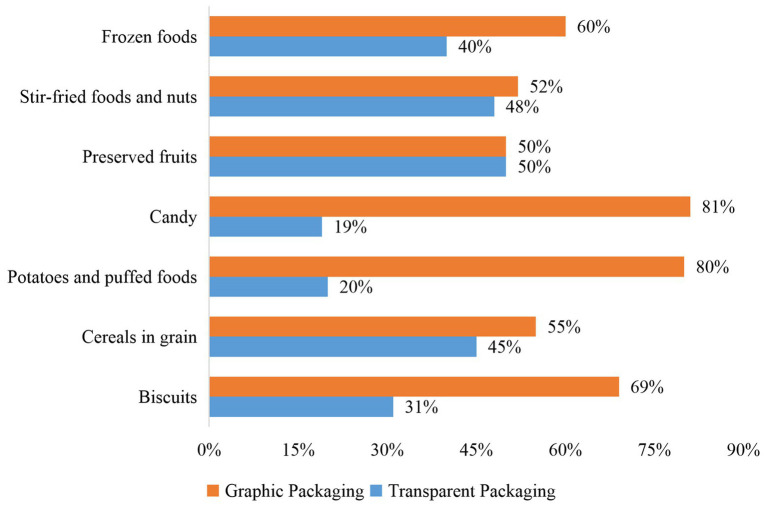
The use of transparent and graphic packaging in different food categories. Proportion of graphic or transparent packaging in one food category = Number of graphic or transparent packaging for the observed food category/Total number of graphic and transparent packaging for that observed food category.

A pilot study was performed before creating the experimental materials. The pilot study investigated the popularity and category of products with transparent packaging in markets in Xi’an. Three off-line supermarkets (Vanguard, Wal-Mart, Yonghui) were visited to survey the transparent materials used to package food products. The survey found that the packaging materials of prepackaged food on the shelves were divided into three categories: box, bag, and can. Of the 170 food brands surveyed, 70.6% of them used bag packaging, 17.1% packaged their products in cans, and 12.3% of them used boxes. Since bags are the most commonly used packaging in prepackaged foods, all food packaging pictures in this study were designed on bags. The pilot study also showed that transparent packaging was frequently used for biscuits, cereals, grain products, potatoes and puffed grain foods, candy products, preserved fruits and other fruit products, stir-fried food, nut products, and frozen foods (see Figure 1). Moreover, our online survey based on a large e-commerce platform in China showed that nut products, preserved fruits, and instant cereals were the three most popular foods for sale. Thus, these three categories were selected to create the experimental materials.

**Figure 2:**
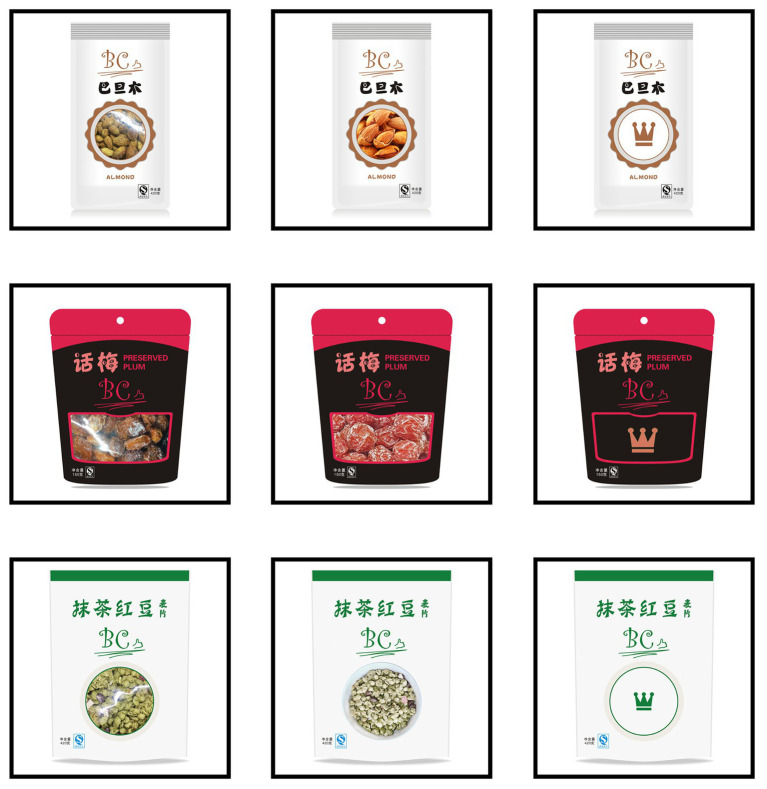
Examples of stimuli used in experiment. First column: transparent window packaging; second column: graphic window packaging; last column: baseline window packaging. Top row: nuts; middle row: preserved fruits; bottom row: instant cereals.

Each product category contains nine different foods, forming a total of 27 food products included in the current study. Adobe Photoshop CS6 software was used to create pictures of experimental food packaging. Each packaging picture contains elements such as the trademark, food name, a picture or transparent window, certification mark, and food net content. Each food was packaged in three different ways: transparent window packaging (*transparent*), graphic window packaging (*graphic*), and baseline window packaging (*baseline*). To avoid the influence of food trademarks on the subjects, all foods were named with a nonexistent brand that the subjects had never seen before (Simmonds et al., 2018b). In order to reduce the visual differences of the three versions of packaging, we matched the position and size of food trademarks and windows, which were also based on actual products in supermarkets to increase ecological validity. Finally, 81 food images were created as stimuli in the formal experiment (see Figure 2 for an illustration of experimental stimuli).

### Apparatus

The stimuli were presented on a 24-inch LCD monitor (ASUS VG248QE) with a resolution of 1920 × 1080 pixels and a refresh rate of 144 Hz. The distance between the participants’ eyes and the screen was 62 cm. Each packaging image occupied about 24 cm vertically and 20 cm horizontally on the screen. Participants’ eye movements were recorded by an EyeLink 1000 Plus (SR Research Ltd., Ontario, Canada) eye tracker with a sample rate of 1,000 Hz. A chin rest was used to reduce head movements. Since both eyes fixate on the same spot, recording one eye is sufficient. Experimental data were collected and processed by the Experiment Builder and Data Viewer software.

### Procedure

After arriving at the lab, participants were asked to rate their hunger levels (1 = not hungry at all, 7 = very hungry) and their desire to eat (1 = not strong at all, 7 = very strong) using a seven-point Likert scale. If a participant’s score was higher than 4, they were asked to eat snacks that were unrelated to the experimental stimuli. When asked again, the participant rated their hunger and desire to eat as less than 4 after eating the snacks. Single factor Latin square experimental design was adopted for the three package types (i.e., transparent window packaging, graphic window packaging, and baseline window packaging). There were three sets of trials, and each set contained 27 trials, which included different food packaging. Participants were randomly assigned to each set.

**Figure 3:**
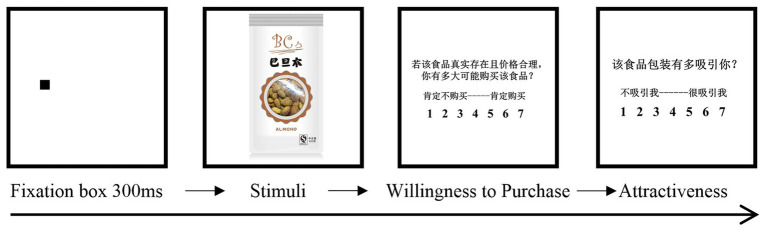
A brief demonstration of experiment procedure.

During the experimental stage, the researcher briefly introduced the overall procedure and experimental equipment to the participants, and then carried out a five-point calibration. After successful calibration, participants finished three practice trials to familiarize the experimental procedure. None of the stimuli used in the practice sessions appeared in the formal experiment. The participants then performed the formal experiment with 27 trials (see Figure 3 for a demonstration). After participants focused on a fixation cross for 300 ms, the packaging images appeared, and participants were asked to view the images freely, pressing the space bar when they were ready to proceed to the next stage. Participants were then asked to estimate their willingness to purchase the product (i.e., *How likely would you be to buy this food, assuming it was available and at a reasonable price?*) and the product’s visual attractiveness (i.e., *How attractive is the packaging to you?*) of each packaging image, using seven-point Likert scales.

### Data Analysis

The main region of interest (ROI) is the window region for each type of packaging. Dependent variables included two perceptual measures (willingness to purchase and packaging attractiveness), and the following three eye movement measures for each ROI. *Time to first fixation* (TTFF; i.e., latent time to first fixate an interest area Ma and Zhuang, 2020) was selected as one of the main indicators of attention capture by salient features. The shorter the time to the first fixation was, the more the salience effect was apparent for the observed area of interest. We also selected two most commonly used eye movement measures: *total time* (TT; i.e., the sum of the duration across all fixations that fall in the current interest area) and *number of fixations* (NF; i.e., total number of fixations falling in the interest area) to reveal the total attention interest in the corresponding region (Wedel and Pieters, 2007; Holmqvist et al., 2011). The extreme value (±2.5 *SD*) of the concerned eye movement measures was excluded. More than 97.6% of data were kept for final analyses. ANOVA was used to analyze the variance of all dependent variables, and Bonferroni corrections were used for paired comparisons. Spearman’s correlation coefficient was used to test the correlation between the eye movement indexes and behavior indexes with SPSS 22.0.

## Results

### Eye-Tracking Measures

#### Time to First Fixation

Detailed eye movement measures and the results of paired comparisons are presented in Table 1. Time to first fixation did not show significant main effects for nuts, *F*(2, 42) = 0.40, *p* = 0.675, and instant cereals, *F*(2, 42) = 1.29, *p* = 0.286. However, for preserved fruits, there were significant differences among different package types, *F*(2, 42) = 4.24, *p* = 0.021. The times to first fixation on the window region in the *“transparent”* condition were significantly shorter than the *“baseline”* condition. This result meant that the transparent window in preserved fruit packaging could capture participants’ attention more quickly.

**Table 1:** Eye movement measures for the three packaging types in each food category.

		Time to first fixation (ms)		Total time (ms)		Number of fixations	
Food	Packaging	*M*	*SD*	*M*	*SD*	*M*	*SD*
Nuts	Transparent	5,052^a^	2,514	2,904^a^	1,306	10.6^a^	4.5
	Graphic	4,915^a^	2,590	2,250^a^	939	7.3^b^	2.7
	Baseline	4,189^a^	3,374	922^b^	532	4.2^c^	2.1
Preserved fruits	Transparent	4,229^b^	2,223	2,007^a^	979	6.9^a^	2.8
	Graphic	5,761^a, b^	2,867	2,335^a^	1,061	8.8^a^	3.9
	Baseline	7,259^a^	3,353	458^b^	314	2.1^b^	1.2
Instant cereals	Transparent	6,282^a^	5,781	2,492^a^	1,177	8.3^a^	3.8
	Graphic	4,420^a^	2,171	1,894^a^	1,102	6.4^a, b^	3.1
	Baseline	7,096^a^	5,254	849^b^	367	3.8^b^	1.6

For a specific eye movement measure, mean values within each food category with different superscript letters are significantly different according to the results of paired comparison with Bonferroni correction (*p* < 0.05).

#### Total Time

The main effects of package type for total time were significant for nuts, *F*(2, 42) = 15.97, *p* < 0.001, preserved fruits, *F*(2, 42) = 20.71, *p* < 0.001, and instant cereals, *F*(2, 42) = 11.39, *p* < 0.001. Paired comparison showed that for the three food categories, the total time in the *“transparent”* conditions (*ps* < 0.001) and *“graphic”* conditions (*ps* < 0.014) were significantly longer than that in the *“baseline”* condition. There was no significant difference between the *“transparent”* and *“graphic”* conditions, *ps* > 0.223.

#### Number of Fixations

The number of fixations also showed significant main effects for nuts, *F*(2, 42) = 14.62, *p* < 0.001, preserved fruits, *F*(2, 42) = 22.21, *p* < 0.001, and instant cereals, *F*(2, 42) = 8.75, *p* = 0.001. Paired comparisons showed that number of fixations in the *“transparent”* conditions (*ps* < 0.001) and *“graphic”* conditions (nuts: *p* = 0.037, preserved fruits, *p* < 0.001, and instant cereals: *p* = 0.062) were significantly more than the *“baseline”* conditions. For nuts, the number of fixations in the *“transparent”* condition was significantly more than the *“graphic”* condition (*p* = 0.024). However, there were no significant differences between the *“transparent”* and *“graphic”* conditions in the other two kinds of foods.

### Perceptual Measures

#### Willingness to Purchase

Descriptive assessment data and the results of paired comparisons are presented in Table 2. For nuts, the main effect of package type was significant, *F*(2, 42) = 3.95, *p* = 0.027. Paired comparison revealed that the willingness to purchase in the *“transparent”* condition was significantly higher than the *“baseline”* condition (*p* = 0.048). Though the main effects of package type for the preserved fruits and instant cereals were insignificant, descriptive statistics showed that the participants were more willing to buy the products with graphic or transparent windows.

**Table 2:** Perceptual measures for the three packaging types in each food category.

		Willingness to purchase		Packaging attractiveness	
Food	Packaging	*M*	*SD*	*M*	*SD*
Nuts	Transparent	4.82^a^	0.90	4.43^a^	0.80
	Graphic	4.76^a, b^	0.89	4.71^a^	0.92
	Baseline	3.93^b^	1.11	3.42^b^	1.24
Preserved fruits	Transparent	4.73^a^	0.86	4.26^a, b^	1.06
	Graphic	4.93^a^	0.85	5.00^a^	0.87
	Baseline	4.20^a^	1.06	3.92^b^	1.07
Instant cereals	Transparent	4.08^a^	1.43	3.90^a^	1.36
	Graphic	4.32^a^	1.10	4.05^a^	1.05
	Baseline	3.34^a^	0.74	2.77^b^	0.93

Mean values within each food category with different superscript letters are significantly different according to results of paired comparison with Bonferroni correction (*p* < 0.05).

#### Packaging Attractiveness

The main effects of package type on packaging attractiveness were significant for nuts, *F*(2, 42) = 6.85, *p* = 0.003, preserved fruits, *F*(2, 42) = 4.53, *p* = 0.017, and instant cereals, *F*(2, 42) = 5.80, *p* = 0.006. For nuts and instant cereals, both the *“transparent”* stimuli (*ps* < 0.027) and *“graphic”* stimuli (*ps* < 0.010) were rated as more attractive than the *“baseline”* stimuli, while there were no differences between the *“transparent”* and *“graphic”* conditions (*ps* > 0.155). For preserved fruits, only the *“graphic”* stimuli were rated as more attractive than the *“baseline”* stimuli (*p* = 0.016). Descriptive statistics also revealed that the graphic window packaging was the most attractive one for all the three kinds of food category.

#### Correlation Between Eye Movements and Perceptual Measures

Correlations between eye movements and perceptual measures in different packaging types under each food category were analyzed though the Spearman’s correlation (see Table 3). For preserved fruits, eye movement measures such as *total time* and *number of fixations* were significantly positively correlated with the scores of packaging attractiveness and willingness to purchase, but the other correlations were insignificant. There were also significantly positive correlations between the scores of packaging attractiveness and willingness to purchase (nuts: *r* = 0.88, *p* < 0.001; preserved fruits: *r* = 0.83, *p* < 0.001; and instant cereals: *r* = 0.94, *p* < 0.001). Therefore, packaging attractiveness, instead of eye movement measuring, is more appropriate to predict willingness to purchase.

**Table 3:** Spearman’s correlation coefficients between eye movements and perceptual measures.

	Willingness to purchase			Packaging attractiveness		
	Nuts	Preserved fruits	Instant cereals	Nuts	Preserved fruits	Instant cereals
Time to first fixation	0.13	0.25	0.05	0.13	0.12	0.07
Total time	−0.03	0.31^*^	0.04	0.03	0.41^**^	0.14
Number of fixations	−0.05	0.33^*^	0.01	0.00	0.43^**^	0.10

* Correlation is significant at the 0.05 level.

** Correlation is significant at the 0.01 level.

## Discussion

The present study used an objective eye-tracking approach to examine how packaging transparency and product category affected consumer attention and purchase behavior. The eye-tracking method overcame the limitation of the subjective evaluation method in assessing a product’s attraction and consumers’ attention toward it (Deng and Srinivasan, 2013; Simmonds et al., 2018a,b, 2019), thus providing more direct evidence to test the salience effect hypothesis. The results of eye-tracking data showed that the salience effect was modulated by product category. Additionally, the results showed that, out of the three product categories tested, transparent window packaging attracted consumer attention most quickly for preserved fruit. In general, transparent window packaging and graphic window packaging capture consumers’ attention better, resulting in longer total time and a larger number of fixations than the baseline condition. Furthermore, the perceptual measures revealed that the willingness to purchase was strongly related to the attractiveness of food packaging for all three food categories, but only had a significant correlation with fixation measures for preserved fruit. Based on these findings, we will discuss the relationship between packaging design and the salience effect, as well as the factors influencing consumers’ purchase intention.

### Salience Potentially Unique to Transparent Window Packaging

In previous studies, according to the salience effect hypothesis, researchers proposed that packaging with transparent elements would always stand out to consumers compared with opaque packaging, and attracted consumers to eat more, or promoted their purchase behavior (Deng and Srinivasan, 2013; Simmonds et al., 2018b). However, using the eye-tracking approach is necessary to definitively ascertain if packaging with transparent elements really captures consumer attention. Generally, consumers tend to look at the top half of a novel product’s packaging (Juravle et al., 2015). However, even a potentially preset scanpath does not influence the comparison of packaging attraction for different packaging types in the current within-subject design experiment.

*Time to first fixation* was used as one of the main indicators to investigate the salience effect in this study. The data showed that only preserved fruits had a significant main effect and whose transparent window packaging successfully attracted consumer attention. For nuts and instant cereals, however, even the descriptive statistics did not show a shorter time to first fixation for product packaging with a transparent window. These data were partially inconsistent with the prediction of the salience effect hypothesis (Spence et al., 2016), which might be caused by the food categories we selected. The foods in this study were selected according to the utilization rate of transparent packaging and food sales volume, which meant that participants had corresponding purchasing experience or a personal preference for these foods. By involving personal expectations, their gaze behaviors were not only affected by the stimulus’ physical characteristics (bottom-up) but also by the participants’ subjective expectations (top-down). Therefore, the early-stage measure of *time to first fixation* may reflect the interaction between the participants’ bottom-up and top-down processing of the packaging pictures.

The later-stage measures, such as *total time* and *number of fixations*, always reflected sustained attention. A number of studies have shown that visually salient stimulus not only captured participants’ attention quickly but also retained longer processing time (Shimojo et al., 2003; Bialkova and van Trijp, 2011; Milosavljevic et al., 2012; Orquin and Mueller Loose, 2013). In this study, *total time* and *number of fixations* of the transparent and graphic window packaging of all three food categories were higher than the baseline window packaging. Also, there was no statistically significant difference between the transparent and graphic window packaging in any food category, except the number of fixations in nuts packaging. These results indicate that showing food information, whether the actual food, or a picture of it, can stand out and retain consumers’ attention, thus H1 is partially verified. Previous studies emphasized the salience effect of transparent packaging but ignored the effects of graphic packaging, which may be related to experimental materials. Deng and Srinivasan (2013) did not involve the category of graphic window packaging in their study. Simmonds et al. (2018b) included graphic window packaging, but they only selected one product out of each food category, thus the generalizability of the transparent packaging effect needs to be further examined. The materials used in the present study consist of nine types of food for each food category, which can provide higher ecological and statistical validity. The data suggest that salience is not limited to transparent window packaging because food-related graphic window packaging can also capture consumers’ attention.

### Attractiveness Influences the Willingness to Purchase

Shimojo et al. (2003) proposed a dual-contribution model in which cognitive assessment systems and orienting behavior structures simultaneously influence people’s preferential decision-making behavior, through facial attractiveness decision experiments. When a stimulus attracts our attention and produces a preference to gaze it, the preference will lead to more exposure to the stimulus, which creates an increased preference. Preference in turn increases our gaze behavior, so as to continuously strengthen our perception of stimulus to influence our decisions (Shimojo et al., 2003; Simion and Shimojo, 2006). This model provides a plausible explanation for the mechanism underlying the transparent window salience effect. The explanation is that when consumers are exposed to the food through a transparent window, the food captures our attention, which seemly produces a gaze preference for the transparent window packaging compared with other packaging. This preference may make consumers pay more attention to transparent window packaging, and the increase in gaze time translates into a higher preference, which could lead to increased willingness to purchase the food in that packaging. Therefore, *total time* and *number of fixations* should have a significant positive correlation with the willingness to purchase. However, the results of this study show that only *total time* and *number of fixations* of preserved fruits were significantly and positively correlated with purchase intention. For nuts and instant cereals, none of the correlations between fixation patterns and willingness to purchase were statistically significant. These results were *inconsistent* with H2. This means that long gaze duration does not necessarily lead to a final purchase decision (see also Balcombe et al., 2017; Wolf et al., 2019). Wolf et al. (2019) found that the relationship between gaze time and three options [i.e., the exclusive evaluation task contains rejection, deferment, and inclusion; the non-exclusive evaluation task contains 1 (“not at all”), 2 (neutral), and 3 (“very much”)] from participants who presented an inverted U-shaped trend. Therefore, the gaze may not necessarily lead to more liking but contribute to the evaluative processing by integrating extra information. As Wolf et al. (2019) suggested, the prolonged viewing time for the middle category may reflect doubt or uncertainty during the evaluative processing, potentially with an increased effort of information integration before reaching a conclusion.

In this study, there was a significant positive correlation between perceived packaging attractiveness and a willingness to purchase. After the formal experiment, when participants were asked why they did or did not want to buy the product, almost all of them mentioned their impressions of the packaging. We speculate that consumers’ willingness to purchase is likely to be affected by the aesthetic perception of packaging. The correlation analyses also revealed that there was a significant positive correlation between packaging attractiveness and willingness to purchase. Simmonds et al. (2018b) proposed that the packaging attractiveness was a necessary premise of supporting the salience effect of transparent window packaging. The authors argued that transparent window packaging could enhance consumers’ purchase intention by highlighting the food inside, and attractive transparent window packaging can make this effect more notable. However, the results of the current study suggest that packaging attractiveness appears to work at a more general level. Showing the food in a concrete form (i.e., presenting actual foods through transparent windows or displaying food images on graphic windows) to consumers can indeed attract consumers’ attention effectively. However, the attractiveness of food packaging most significantly promotes consumers’ willingness to purchase the product.

### Salience of Different Food Categories

Combined with eye movement and perceptual measures, this study further revealed that product category regulated the effect of packaging type on consumer attention. That means consumers may have different gaze patterns for different products. For nuts, most are shelled, and the difference between the actual food inside the packaging and the food image is slight. The data showed that there were no statistical differences between *“transparent”* and *“graphic”* stimuli for all the measures except for *number of fixations*, which suggests that both transparent and graphic window packaging have similar salience effects for nuts. Therefore, transparent window packaging and graphic window packaging can efficiently capture consumers’ attention and increase product attractiveness.

Participants preferred the graphic window packaging on preserved fruits more than the transparent window packaging according to the perceptual results, although the preference was slight. In combination with participants’ feedback after the experiment, we found that most types of preserved fruits in this study were often coated in sugar or honey. These sticky coatings would smear against the inner transparent window of the packaging and reduce participants’ appetite. Therefore, when participants saw the sugar‐ or honey-coated preserved fruits through the transparent window, they would avoid the package. Most preserved fruits are sticky or frosted, and thus graphic window packaging is more suitable to attract consumers to these products.

In their study, Simmonds et al. (2018b) also inspected instant cereals. Although the pictures of the cereal packaging in this study were similar to their experimental materials, there are several differences. Only one food for each food category was used in their study, and the baseline was a blank condition. In this study, nine types of foods for each category were selected, and the baseline was size-matched non-food graphic objects. Moreover, Simmonds et al. (2018b) asked participants to sort different packaging types presented at the same time according to participants’ judgments. This method was more likely to induce significant differences between the transparent and graphic conditions than the one-by-one estimation method used in the present study. These differences may contribute to the different conclusion they arrived at, where the salience effect only applied to transparent conditions. The present study revealed salience effects for both transparent and graphic conditions.

### Limitations and Future Directions

The present study has several limitations. First, all conclusions of this study are based on Chinese college students and packaging types used in the Chinese market. Future research could explore the salience effect in food packaging across varied age groups and different cultures. Second, this study only involves three food categories, and all of them belong to “leisure food.” Future studies can enrich and subdivide food categories (such as fresh food and cooked food) to investigate the generalization of the salience effect. Third, whether the crown in baseline packaging played as a novel stimulus in a way for some participants was not clear, which should be a more concern in future researches. Fourth, this study adopts traditional laboratory methods, which may be not enough to measure ecological validity. Besides, participants in the current study had known their task to assess willingness to purchase, which might drive their eye movement behavior. Future studies should use more ecological tasks as that in real-life shopping. Finally, in reality, transparent packaging not only allows the real food to be seen through a clear window but also allows consumers to get a full view of the food by shaking the package. However, the latter function is lost in the transparent packing images when viewed on a screen. This may be one of the reasons that diminished the advantages of transparent packaging in this study. Thus, researchers can use field experiments or VR technology to further improve the ecological validity of corresponding studies.

## Conclusion

1. Both transparent and attractively graphic window packaging capture more attention compared to the baseline window packaging, but a transparent window does not always gain more benefit than an attractive image.
2. Transparent or graphic window packaging is recommended for the three studied foods, and graphic window packaging is specifically recommended for preserved fruits. Food manufacturers should pay attention to sensory studies to improve packaging design considering food categories.
3. Attractive packaging with salient elements helps to enhance consumers’ willingness to purchase.

## Data Availability Statement

The raw data supporting the conclusions of this article will be made available by the authors, without undue reservation.

## Ethics Statement

The studies involving human participants were reviewed and approved by Academic Committee of Shaanxi Normal University. The patients/participants provided their written informed consent to participate in this study.

## Author Contributions

All authors contributed to the design of the study. XM conducted the data collection and analyses for the study. XM and GM programmed the experiments. XM, XZ, and GM wrote the manuscript. All authors contributed to the article and approved the submitted version.

### Conflict of Interest

The authors declare that the research was conducted in the absence of any commercial or financial relationships that could be construed as a potential conflict of interest.
